# Acute myocardial infarction, associated with the use of a synthetic adamantyl-cannabinoid: a case report

**DOI:** 10.1186/s40360-016-0045-1

**Published:** 2016-01-16

**Authors:** Graham McIlroy, Loretta Ford, Jawad M. Khan

**Affiliations:** Department of Cardiology, Sandwell and West Birmingham Hospitals NHS Trust, Dudley Road, Birmingham, B18 7QH UK; Department of Clinical Biochemistry, Sandwell and West Birmingham Hospitals NHS Trust, Dudley Road, Birmingham, B18 7QH UK

**Keywords:** Myocardial infarction, Novel psychoactive compounds, Synthetic cannabinoid

## Abstract

**Background:**

“Legal highs” are novel psychoactive substances that have evaded statutory control. Synthetic cannabinoid compounds with adamantane moieties have recently been identified, which have high potency at target receptors and are undetectable on conventional toxicology testing. However, little is known about any harmful effects, and their potential to cause serious ill health. We describe a case of myocardial infarction following the use of this class of drug.

**Case presentation:**

We report the case of a 39-year-old man admitted after an out-of-hospital cardiac arrest, in whom ECG and elevated cardiac enzymes confirmed ST-elevation myocardial infarction. Normal coronary perfusion was restored after thrombectomy and coronary artery stenting. In the hours preceding his admission, the patient is known to have consumed the legal high product “Black Mamba”. Subsequent urine testing confirmed the presence of an adamantyl-group synthetic cannabinoid, whilst cannabis, cocaine, amphetamines and other drugs of abuse were not detected.

**Conclusion:**

The use of legal highs is being increasingly recognised, but the chemical compositions and physiological effects of these drugs are poorly characterised and are continually changing. Synthetic cannabinoids, rarely identified on toxicological testing, can be linked to serious adverse cardiovascular events. This case highlights the importance of testing for novel psychoactive compounds, and recognising their potential to cause life-threatening conditions.

## Background

Recreational use of cannabis dates back centuries. More recently, herbal mixtures have emerged that produce similar effects to cannabis when smoked. Sold under various names, including Spice, K2 and Black Mamba, these preparations can contain synthetically-derived compounds with high affinity for endogenous cannabinoid CB receptors [1].

Novel psychoactive compounds are constantly being developed and later prohibited in a cat-and-mouse game between the legal high manufacturers and governments. Synthetic cannabinoids represent an evolving drug class that are sold openly (although frequently labelled “not for human consumption”), produce stimulant effects in those that smoke them, and are often undetectable on routine toxicology testing.

However, because of their unregulated development, synthetic cannabinoids can have potentially serious side effects, putting those that use them at an unknown risk of harm. Here, we report a case of myocardial infarction and cardiac arrest associated with a new class of adamantyl-group synthetic cannabinoid, confirmed by urine drug testing.

## Case presentation

A 39-year-old man presented to the emergency department after a witnessed collapse and cardiac arrest. He was Caucasian, with a body mass index of 28.8 kg/m^2^, and a smoker. His past medical history consisted of depression and a single episode of deep venous thrombosis of the calf. There was no personal or family history of cardiac disease, he was neither diabetic nor hypertensive, and had a normal serum cholesterol level (2.5 mmol/l). Although he initially denied illicit drug use, a third party confirmed he had smoked “Black Mamba” within three hours of the onset of his symptoms.

**Fig. 1:**
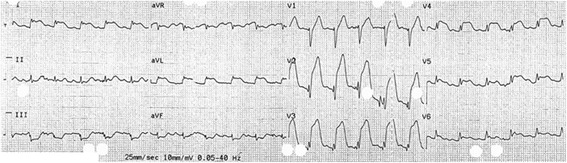
The patient’s ECG, recorded en route to the emergency department, showing ST elevation in anterolateral leads

He initially reported left-sided chest pain, associated with dizziness and dyspnoea. Minutes later, the patient collapsed, cardiac output became undetectable, and cardio-pulmonary resuscitation was commenced. Out-of-hospital cardiac monitoring revealed ventricular fibrillation, and spontaneous circulation was restored after delivery of four direct current shocks. During transfer to hospital, ECG demonstrated ST elevation in antero-lateral leads (Fig. 1), and the patient was admitted directly for cardiac catheterisation. The serum Troponin-T level was recorded at 4398 ng/L (normal range <14 ng/L on high-sensitivity Troponin-T), confirming the diagnosis of myocardial infarction.

Coronary angiography revealed an ostial occlusion to the left anterior descending artery, which was treated by thrombectomy. There was some residual stenosis, which could have represented mild coronary artery disease or persistent vasospasm. As it did not respond to nitrate treatment, a Xience drug-eluting stent (Abbott) was placed. The left circumflex and right coronary arteries were angiographically normal, and there were only mild irregularities in an intermediate artery. The patient experienced no further symptoms of cardiac ischemia, and the ST elevation resolved after percutaneous coronary intervention. He was discharged with anti-platelet therapy (ticagrelor 90 mg twice daily for one year, aspirin 75 mg daily for life) and secondary prevention for coronary artery disease (atorvastatin 80 mg daily, bisoprolol 2.5 mg daily and perindopril 2 mg daily).

Urine toxicology tested positive for opiates and benzodiazepines (both given on admission to hospital), and also for adamantyl-group synthetic cannabinoids. Other drugs of abuse were not detected, including cannabis, cocaine and amphetamines. Drugs were assayed by liquid tandem mass spectrometry (UPLC-MS/MS), and confirmed by time-of-flight mass spectroscopy.

## Discussion

In England and Wales, cannabis is consistently the most frequently used illegal drug [2]. Although relatively uncommon, cannabis use is associated with a number of serious cardiovascular conditions, including myocardial infarction and stroke [3], with cases of both coronary artery thrombosis and vasospasm reported [4, 5]. Synthetic cannabinoid use has been associated with admission to hospital for a range of symptoms, including anxiety, seizure, vomiting, and chest pain [6, 7]. ECG changes are rarely seen after synthetic cannabinoid use, but possible mechanisms for increasing the risk of myocardial infarction include tachycardia, hypertension, and metabolic disturbances such at hypokalaemia [8]. Although most toxicities are classed as minor or moderate [9], more synthetic cannabinoid users seek medical attention than would be expected for cannabis [10]. The increased risk of adverse reaction to synthetic cannabinoid, compared to cannabis, could be due to higher potency at CB receptors, or to uncharacterised interactions with other endogenous targets. Moreover, with the diversity of molecular moieties seen on synthetic cannabinoids, it is possible that the different classes have distinct target profiles.

Synthetic cannabinoid use has previously been associated with acute myocardial infarction, in both adults and children [11–14]. However, in many of these reports, the self-reported use of synthetic cannabinoid products is not confirmed by biochemical testing, and some patients also tested positive for cannabis. Here, we describe a patient whose reported drug use was confirmed by urine toxicology testing, which also ruled out use of cannabis and other drugs of abuse. Animal studies have recorded physiological effects of adamantyl-cannabinoids from one to six hours after administration, and a human study found metabolite excretion peaked five to seven hours after self-administration and was still detectable up to 160 hours later [15–17]. Although inhaling synthetic cannabinoids results in unpredictable doses [17], the details of our case are consistent with the known pharmacological properties of these substances. Coronary angiography showed mild residual irregularities after thrombectomy, and given a personal history of deep venous thrombosis, it is possible that this patient was particularly vulnerable to a cardiovascular event. However, his 10-year QRISK2 score (ClinRisk Ltd.) was 2.6 %, therefore the major contributor to this event is most likely the consumption of the adamantyl-cannabinoid in the hours before his presentation.

We report a case of acute myocardial infarction associated with use of an adamantyl-cannabinoid. Adamantane moieties have recently been found in synthetic cannabinoid compounds, and they produce potent CB receptor ligands [18]. The Clinical Biochemistry Department of our NHS Trust, closely linked to the UK National Poisons Information Service, continually screens samples received for toxicology testing, as well as legal high samples seized by UK Trading Standards and from prisons. Our testing has found that adamantyl-cannabinoids are currently the most popular class of synthetic cannabinoid on sale in the UK, particularly AKB-48, 5 F-AKB-48 and STS-135. Our drugs of abuse screen, carried out on urine samples, yields a positive result for adamantyl-cannabinoids if any of these compounds are detected [19].

Most of the previous reports of acute myocardial infarction were associated with the JWH class of synthetic cannabinoid. However, the trend in Europe has been away from this older class of drug, likely in response to its scheduling as illegal, towards more potent compounds [9]. Consistent with this, our laboratory now rarely detects JWH-class synthetic cannabinoids.

Importantly, the street name of the legal high does not necessarily indicate which psychoactive compound is present. A recent outbreak of neurotoxicity and cardiotoxicity in the US was attributed to a synthetic cannabinoid known locally as “Black Mamba” [20]. However, the compound identified from these patients is chemically distinct from the “Black Mamba” detected in our patient, as it lacks the characteristic adamantane moiety. It is noteworthy that the adamantyl-cannabinoid AKB-48 has been scheduled as illegal in the US [21], whilst it currently remains unscheduled in the UK. This reflects the ongoing evolution of legal highs, driven by consumer demand for the drugs and statutory bodies criminalising their use.

## Conclusions

The legal high market is buoyant and rapidly evolving. Whilst many illegal and controlled pharmaceutical drugs can be identified with toxicology testing, novel psychoactive substances are often undetectable on routine testing. Their prevalence is therefore likely to be under-reported, and their precise chemical composition unknown. However, use of synthetic cannabinoids exposes people to the risk of serious harm, including acute myocardial infarction. It is important to identify when patients have consumed psychoactive substances, both to manage any complications that arise, and also to inform the wider public health debate.

### Consent

Written informed consent was obtained from the patient for publication of this case report and any accompanying images. A copy of the written consent is available for review by the Editor of this journal.

## Abbreviations

ECG: Electrocardiogram
NHS: National health service
